# Antioxidant Activities and Mechanisms of Tomentosin in Human Keratinocytes

**DOI:** 10.3390/antiox11050990

**Published:** 2022-05-18

**Authors:** Seyoung Yang, See-Hyoung Park, Sae Woong Oh, Kitae Kwon, Eunbi Yu, Chae Won Lee, Youn Kyoung Son, Changmu Kim, Byoung-Hee Lee, Jae Youl Cho, Youn-Jung Kim, Jongsung Lee

**Affiliations:** 1 Molecular Dermatology Laboratory, Department of Integrative Biotechnology, College of Biotechnology and Bioengineering, Sungkyunkwan University, Suwon City 16419, Gyunggi Do, Korea; yy1771@skku.edu (S.Y.); hanzeeoo@skku.edu (S.W.O.); wesdwe1@skku.edu (K.K.); yuebi95@skku.edu (E.Y.); 2 Department of Bio and Chemical Engineering, Hongik University, Sejong City 30016, Korea; shpark74@hongik.ac.kr; 3 National Institute of Biological Resources, Environmental Research Complex, Incheon 22689, Korea; chaewon326@korea.kr (C.W.L.); sophy004@korea.kr (Y.K.S.); snubull@korea.kr (C.K.); dpt510@korea.kr (B.-H.L.); 4 Molecular Immunology Laboratory, Department of Integrative Biotechnology, College of Biotechnology and Bioengineering, Sungkyunkwan University, Suwon City 16419, Gyunggi Do, Korea; 5 Department of Marine Sciences, Incheon National University, Incheon 22012, Korea

**Keywords:** tomentosin, human keratinocytes, ROS, p38 MAPK, JNK, Nrf2, AhR

## Abstract

Tomentosin, one of natural sesquiterpene lactones sourced from *Inula viscosa* L., exerts therapeutic effects in various cell types. Here, we investigated the antioxidant activities and the underlying action mechanisms of tomentosin in HaCaT cells (a human keratinocyte cell line). Specifically, we examined the involvement of tomentosin in aryl hydrocarbon receptor (AhR) and nuclear factor erythroid 2-related factor 2 (Nrf2) signaling pathways. Treatment with tomentosin for up to 60 min triggered the production of reactive oxygen species (ROS), whereas treatment for 4 h or longer decreased ROS production. Tomentosin treatment also induced the nuclear translocation of Nrf2 and upregulated the expression of Nrf2 and its target genes. These data indicate that tomentosin induces ROS production at an early stage which activates the Nrf2 pathway by disrupting the Nrf2–Keap1 complex. However, at a later stage, ROS levels were reduced by tomentosin-induced upregulation of antioxidant genes. In addition, tomentosin induced the phosphorylation of mitogen-activated protein kinases (MAPKs) including p38 MAPK and c-Jun *N*-terminal kinase (JNK). SB203580 (a p38 MAPK inhibitor) and SP600125 (a JNK inhibitor) attenuated the tomentosin-induced phosphorylation of Nrf2, suggesting that JNK and p38 MAPK signaling pathways can contribute to the tomentosin-induced Nrf2 activation through phosphorylation of Nrf2. Furthermore, *N*-acetyl-*_L_*-cysteine (NAC) treatment blocked both tomentosin-induced production of ROS and the nuclear translocation of Nrf2. These data suggest that tomentosin-induced Nrf2 signaling is mediated both by tomentosin-induced ROS production and the activation of p38 MAPK and JNK. Moreover, tomentosin inhibited the AhR signaling pathway, as evidenced by the suppression of xenobiotic-response element (XRE) reporter activity and the translocation of AhR into nucleus induced by urban pollutants, especially benzo[a]pyrene. These findings suggest that tomentosin can ameliorate skin damage induced by environmental pollutants.

## 1. Introduction

Reactive oxygen species (ROS) are produced intracellularly during cellular metabolism, mainly in the mitochondria. ROS formation can occur when the cells are exposed to cellular or extracellular stress caused by agents such as xenobiotics, cytokines, ultraviolet (UV) light, and environmental pollutants [1,2,3]. Oxidative stress caused by the accumulation of ROS that outweighs the antioxidant capacity of the cells damages the cells and intracellular macromolecules such as proteins, lipids, and DNA [4]. Moreover, oxidative stress is involved in various diseases such as vitiligo, aging, diabetes, and cancer [5]. In contrast, ROS functions as a second messenger in various signaling cascades. ROS levels below the cellular tolerance threshold activate ROS signaling pathways that regulate ROS homeostasis and prevent cell damage [6].

Nuclear factor erythroid 2-related factor 2 (Nrf2) plays a major role in sensing and eliminating oxidative stress in cells. Nrf2 is a transcription factor that interacts with the antioxidant response element (ARE) and regulates the expression of phase Ⅱ detoxifying and antioxidant enzymes including NAD(P)H-quinone oxidoreductase 1 (NQO1) and heme oxygenase-1 (HO-1) [7]. Under basal conditions, Nrf2 forms a complex with two repressor proteins, namely Cullin 3 (Cul3) and Kelch-like ECH-associated protein 1 (Keap1) in the cytoplasm. However, under oxidative stress conditions, the modification of cysteine residues in Keap1 induces a conformational change in the Keap1 protein which results in the dissociation of Nrf2 from the Nrf2–Keap1–Cul3 complex. Free Nrf2 translocates to the nucleus and heterodimerizes with the small Maf protein. This heterodimer induces the expression of Nrf2 target genes by binding to the ARE region in the promoters of these genes [8].

It has been known that the nuclear translocation and degradation of Nrf2 are modulated by the post-translational modification of Nrf2, especially phosphorylation [9]. The phosphorylation of Nrf2 at Ser-40 by protein kinase C (PKC) leads to the dissociation of the Nrf2–Keap1 complex and prompts the nuclear translocation of Nrf2 [10]. Additionally, mitogen-activated protein kinases (MAPKs) and the phosphatidylinositol 3-kinase (PI3K/AKT) pathway are also known contribute to the phosphorylation and activation of Nrf2 [9].

Benzo[a]pyrene (B[a]P), one of the polycyclic aromatic hydrocarbons, is a commonly occurring environmental pollutant and is classified as a human carcinogen [11]. It is also known to be a component of particulate matter. B[a]P induces the production of pro-inflammatory cytokines and ROS through the aryl hydrocarbon receptor (AhR) signaling pathway [12]. B[a]P binds to AhR in the cytoplasm and induces translocation of AhR into the nucleus. AhR that is activated by B[a]P forms a complex with AhR nuclear translocator (ARNT) in the nucleus. The complex subsequently interacts with the xenobiotic response element (XRE) and upregulates the expression of its target gene, cytochrome P450 1A1 (CYP1A1) [11].

Tomentosin, a natural sesquiterpene lactone, is one of main components of several aromatic medicinal species such as *Inula viscosa* (L.) Aiton [13]. It has been used as a therapeutic agent because it possesses pharmacological activity and exhibits anti-cancer, anti-bacterial, and anti-inflammatory effects [14,15,16,17]. Although the effects of tomentosin have been actively examined in cancer cells, there are no studies on the protective effects of tomentosin in skin tissue. Therefore, in this study, we investigated the antioxidant activities of tomentosin and its mechanisms of action in the HaCaT human keratinocyte cell line.

## 2. Materials and Methods

### 2.1. Reagents and Cell Culture

HaCaT cells (American Type Culture Collections, Manassas, VA, USA) were incubated in Dulbecco’s modified Eagle’s medium (DMEM, SH30243.01, Hyclone, Logan, UT, USA) supplemented with 1% antibiotics (penicillin/streptomycin) and 10% fetal bovine serum (FBS) at 37 °C/5% CO_2_ in a humidified incubator. Tomentosin (purity: ≥90%) was purchased from Ensol Biosciences (ES276-A, Daejeon, Korea) and solubilized in dimethyl sulfoxide (DMSO) (472301, Sigma-Aldrich, St. Louis, MO, USA). A 20 mM stock solution of tomentosin was maintained at −20 °C. Antibodies for c-Jun N-terminal kinase (JNK, sc-572), p-JNK (sc-6254), extracellular signal-regulated kinase (ERK, sc-292838) 1/2, p-ERK 1/2 (Tyr 204, sc-101761), p38 MAPK (sc-535), AhR (sc-133088), CYP1A1 (sc-25304), and lamin B1 (sc-365962) were obtained from Santa Cruz Biotechnology (Santa Cruz, Dallas, TX, USA). Antibodies against p-p38 MAPK (9216S), Akt (9272S), and p-Akt (12694S) were purchased from Cell Signaling Technology (CST, Danvers, MA, USA). Nrf2 (ab137550), p-Nrf2 (ab76026), NQO1 (ab28947), HO-1 (ab13248), Keap1 (ab118285), and α-tubulin (ab7291) antibodies were acquired from Abcam (Abcam, Cambridge, UK). Antibodies against β-actin (A5316), anti-goat IgG (A8919), anti-rabbit immunoglobulin G (IgG) (A0545), and anti-mouse IgG (A9044) were obtained from Sigma-Aldrich (Sigma-Aldrich, St. Louis, MO, USA). Goat anti-rabbit IgG (Alexa fluor 594, ab150084) and Goat anti-mouse IgG (Alexa fluor 488, ab150117) were obtained from Abcam (Abcam, Cambridge, UK). B[a]P (CAS No. 50-32-8, purity 99.9%), urban particulate matter (UPM, NIST1648A) and N-acetyl-L-cysteine (NAC, A7250), SB203580 (p38 MAPK inhibitor, S8307), SP600125 (JNK inhibitor, S5567) were obtained from Sigma-Aldrich (Sigma-Aldrich, St. Louis, MO, USA).

### 2.2. Assay for Cell Viability

The cytotoxicity of tomentosin in HaCaT cells was evaluated using Cell Counting Kit-8 (CCK-8, CK04-11, Dojindo, Japan). HaCaT cells were seeded in 12-well plates and incubated in DMEM in the presence of different concentrations of tomentosin for 24 h. After treatment, CCK-8 (4 μL/well) was added into the wells, and the plates were incubated at 37 °C for 2 h. Then, the cell culture media was transferred to a 96-well plate and the absorbance was measured at 450 nm by a microplate reader (Synergy HTX Multi-Mode Reader, Biotek, VT, USA). Results were verified by repeating the experiment four times.

### 2.3. ARE and XRE Luciferase Reporter Assay and β-Galactosidase Assay

HaCaT cells were seeded in 6-well plates and incubated in DMEM at 37 °C overnight. The cells were transiently co-transfected with 1 μg xenobiotic response element (XRE) (Stratagene, La Jolla, CA, USA) or 1 μg antioxidant response element (ARE)-driven luciferase reporter plasmid (Addgene, Watertown, MA, USA) and 1 μg β-galactosidase plasmid. A total of 5 μg of polyethylenimine (PEI) (23966-2, Polysciences, Inc., Warrington, PA, USA) was used for performing the transfections. After 4 h, the medium was replaced with fresh medium for stabilizing the cells. The transfected cells were incubated with various concentrations of tomentosin in the presence or absence of 3 μM B[a]P or 100 μg/mL UPM (National Institute of Standards and Technology, NIST-1648a, Sigma-Aldrich, St. Louis, MO, USA) for 24 h. The cells were collected with phosphate-buffered saline (PBS) and centrifuged at 16,200× *g* for 5 min. The centrifuged cells were lysed with Reporter Lysis Buffer (E3971, Promega, Madison, WI, USA). The lysates were centrifuged at 12,000× *g* for 3 min at 4 °C, and the supernatant was transferred to 96-well plates. The luciferase and β-galactosidase activities of the supernatants were measured following the manufacturer’s instructions (Promega Corporation). The XRE and ARE promoter activities were expressed as ratios of firefly luciferase activity to β-galactosidase activity. Results were verified by repeating the experiment four times.

### 2.4. Dichlorofluorescin Diacetate (DCFDA) Cellular ROS Detection Assay

ROS production was quantitatively determined both by fluorescence microscopy and by using the DCFDA-cellular ROS detection assay kit (ab113851, Abcam, Cambridge, UK). Cells were plated in 60 mm dishes and 96-well opaque wall plates. The cells were washed three times using PBS and subjected to staining with 20 µM DCFDA in PBS for 20 min at 37 °C under dark conditions. After staining, the cells were washed with PBS again and incubated with 55 µM tert-butyl hydroperoxide (TBHP, 458139, Sigma-Aldrich, St. Louis, MO, USA) solution (positive control). Tomentosin treatment was performed for 24 h before DCFDA incubation or for up to 60 min after DCFDA incubation. DCFDA fluorescence signals were detected at an excitation wavelength of 485 nm and an emission wavelength of 535 nm. The change in fluorescence was expressed as a percentage of the control after background subtraction. Results were verified by repeating the experiment four times.

### 2.5. Diphenyl-1-picrylhydrazyl (DPPH) Radical Scavenging Assay

The free radical scavenging activity of tomentosin was quantitated using 2,2-diphenyl-1-picrylhydrazyl (DPPH) assay. Different concentrations of tomentosin were added separately to 0.15 mM DPPH (D9132, Sigma-Aldrich, St. Louis, MO, USA) and incubated for 10 min at room temperature. The absorbance of this mixture was then measured at 517 nm. Then, 100 μM ascorbic acid was introduced as the positive control. Results were verified by repeating the experiment four times. The percent radical scavenging activity was calculated using the following equation:

% Radical scavenging activity = [(Absorbance of control − Absorbance of sample)/Absorbance of control] × 100

### 2.6. Real-Time RT-PCR Analysis of mRNA Levels

Total RNA was extracted from cells by QIAzol lysis reagent (79306, Qiagen, Hilden, Germany) following the manufacturer’s protocols and maintained at −70 °C until use. cDNA was made from total RNA (2 μg) using TOPscript™RT Drymix (RT200, Enzynomics, Daejeon, Korea) following the manufacturer’s protocols. Real-time RT-PCR was conducted using the QuantiSpeed SYBR NO-ROX kit (QS105-10, Phile Korea, Seoul, Korea). Endogenous glyceraldehyde 3-phosphate dehydrogenase (*GAPDH*) (accession number: NM_001256799) was introduced for data normalization. The mRNA levels of the target genes were normalized to the levels observed in controls. Results were confirmed by performing the experiment four times: triplicate samples were included in each experiment. The primer sequences were as follows: *AhR* (accession number: NM_015627): Forward: CGT TTC TTT CAG TAG GGG AGG AT, Reverse: CTT AGG CTC AGC GTC AGT TAC; *HO-1* (accession number: NM_002133): Forward: AAG ACT GCG TTC CTG CTC AAC, Reverse: AAA GCC CTA CAG CAA CTG TCG; *NQO1* (accession number: NM_001025434): Forward: GAA GAG CAC TGA TCG TAC TGG C, Reverse: GGA TAC TGA AAG TTC GCA GGG.

### 2.7. Measurement of Protein Levels Using Western Blot Analysis

HaCaT cells were seeded in 60 mm dishes and treated as per the experimental plan. Protein samples were then prepared from the treated cells. For protein extraction, the cells were collected and centrifuged at 15,000 rpm for 50 min at 4 °C. Cell pellets were lysed using RIPA buffer (150 mM NaCl, 1% sodium deoxycholate, 25 mM Tris-HCl (pH 7.6), 1% NP-40, 0.1% SDS (9806s, CST, Danvers, MA, USA)) including phosphatase and protease inhibitor cocktail (5872s, CST, Danvers, MA, USA). Protein samples were quantified with a BCA assay kit (Thermo Fisher Scientific, Waltham, MA, USA) After that, the protein samples were separated by sodium dodecyl sulfate-polyacrylamide gel electrophoresis, and transferred to polyvinylidene difluoride membranes (162-0177, Bio-Rad, CA, USA). The polyvinylidene difluoride membranes were blocked with BSA and exposed to antibodies. Finally, the proteins were measured using ECL Western Blotting Reagents (170-5061, Bio-Rad, CA, USA). Results were verified by repeating the experiment four times.

### 2.8. Preparation of the Nuclear and Cytoplasmic Cell Fractions

Cytoplasmic and nuclear and cell extracts were fractionated by NE-PER Nuclear and Cytoplasmic Extraction reagents (78833, Thermo Fisher Scientific, Waltham, MA, USA) following the manufacturer’s instructions, and then subjected to Western blot analysis for analyzing target proteins.

### 2.9. Detection of Target Proteins Using Immunocytochemistry

The cells were fixed with 4% paraformaldehyde in PBS for 15 min and permeabilized using 0.01% Tween-20 and 0.1% Triton X-100 for 20 min at room temperature. After blocking the cells with 3% BSA in PBS, the cells were treated with anti-Nrf2 (1:1000; ab76026, Abcam) or anti-AHR (1:500, sc-133088, CST, Danvers, MA, USA) antibodies. The fixed cells were then washed three times and treated with Flamma-594 secondary or Flamma-488 secondary antibodies (Abcam, Cambridge, UK). Subsequently, after counterstaining with Hoechst33342, cells were mounted on slide glasses and observed under a LSM 700 laser scanning confocal microscope (Zeiss, Jena, Germany) with a C-Apochromat 20× objective. Images of the cells were captured uniformly at a set laser power and the mean intensity of the fluorescence signals was determined. The data were analyzed with ZEN 2012 Blue (Zeiss, Jena, Germany) under similar processing parameters. Results were verified by repeating the experiment four times.

### 2.10. Enzyme-Linked Immunosorbent Assay (ELISA) for Target Proteins

An interleukin (IL)-8 ELISA Kit (ab46032, Abcam, Cambridge, UK) was used to quantitate IL-8 levels following the manufacturer’s protocol. The measurement of absorbance was analyzed using a Labsystems Multiskan MS Analyser (Thermo Bio-Analysis Japan, Tokyo, Japan). The results were repeated in three independent experiments.

### 2.11. Statistical Analysis for Data Significance

All results are expressed as mean ± SD and were confirmed in at least three independent experiments. Statistical analysis of the data was conducted using Student’s *t*-test for independent samples. A *p*-value < 0.05 was considered statistically significant.

## 3. Results

### 3.1. Tomentosin Exerts Antioxidant Activity in HaCaT Cells

**Figure 1:**
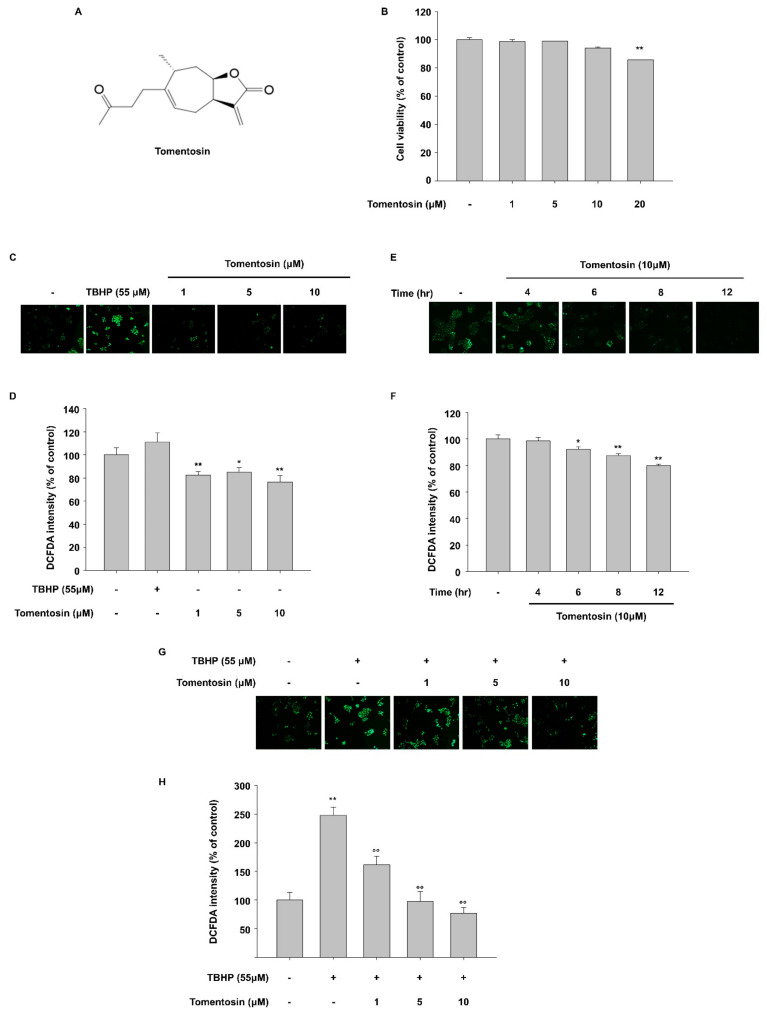
Tomentosin decreases reactive oxygen species (ROS) in a time- and dose-dependent manner in HaCaT cells. (**A**) Chemical structure of tomentosin. (**B**) Cell Counting Kit-8 (CCK-8) was introduced for determining cell viability. HaCaT cells were cultured with the indicated concentrations of tomentosin for 24 h and subjected to CCK-8 analysis. The data are presented as the mean ± SD of triplicates. (**C**,**D**) HaCaT cells were pretreated with tomentosin for 24 h prior to treatment with 20 μM 2′,7′ dichlorofluorescin diacetate (DCFDA) for 20 min, and then subjected to fluorescence microscopy (Original magnification = 10×) (**C**) or microplate-based fluorescence intensity analysis (**D**). The data are presented as the mean ± SD of triplicates. (**E**,**F**) HaCaT cells were pretreated with 10 μM tomentosin for the indicated time periods prior to treatment with 20 μM DCFDA for 20 min and then subjected to fluorescence microscopy (**E**) (Original magnification = 10×) or microplate-based fluorescence intensity analysis (**F**). The data are presented as the mean ± SD of triplicates. (**G**,**H**) HaCaT cells were pretreated with tomentosin for 24 h prior to treatment with 20 μM DCFDA for 20 min. After 20 min, tert-butyl hydroperoxide (TBHP) was added to a concentration of 55 μM and the cells were incubated for 1 h before being subjected to fluorescence microscopy (**G**) (Original magnification = 10×) or fluorescence intensity analysis (**H**). ROS production was assessed using DCFDA. The data are shown as the mean ± SD of triplicates. * *p* < 0.05 vs. untreated group, ** *p* < 0.01 vs. untreated group, ^oo^ *p* < 0.05 vs. TBHP-treated group. TBHP was introduced as a positive control. TBHP, tert-Butyl hydroperoxide.

The tomentosin treatment concentrations were selected based on the cytotoxicity of tomentosin as assessed by the CCK-8 assay. Tomentosin showed no cytotoxicity at 10 μM in HaCaT cells, based on the observation that there was no significant alteration in cell viability (Figure 1B). In addition, tomentosin was reported to increase intracellular ROS levels in various cancer cell types [8,18]. Therefore, we conducted a DCFDA cellular ROS detection assay to investigate the involvement of tomentosin in ROS production in HaCaT cells (human normal keratinocyte cell line). Cells were incubated with different concentrations of tomentosin (1, 5, and 10 μM) for 24 h. As shown in Figure 1C,D, basal ROS production was reduced in tomentosin-treated HaCaT cells in a concentration-dependent manner. Tert-butyl hydroperoxide (TBHP) was introduced as a positive control. In addition, we examined the time point at which tomentosin reduces ROS generation in cells. Cells were incubated with 10 μM tomentosin for different time periods. ROS levels were measured by imaging assays (Figure 1E) and by fluorescence intensity assays (Figure 1F). As shown in Figure 1E,F, tomentosin treatment for >6 h was able to reduce intracellular ROS levels in a time-dependent manner. Furthermore, TBHP-induced ROS production was attenuated by tomentosin treatment (Figure 1G,H). These data indicate that tomentosin has antioxidant activity in a time- and concentration-dependent manner.

### 3.2. Tomentosin Activates the Nrf2 Signaling Pathway

**Figure 2:**
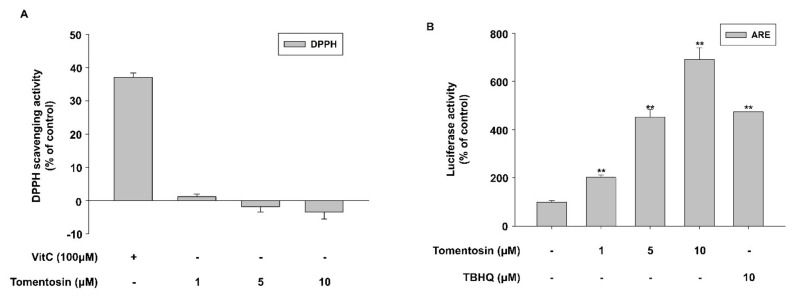

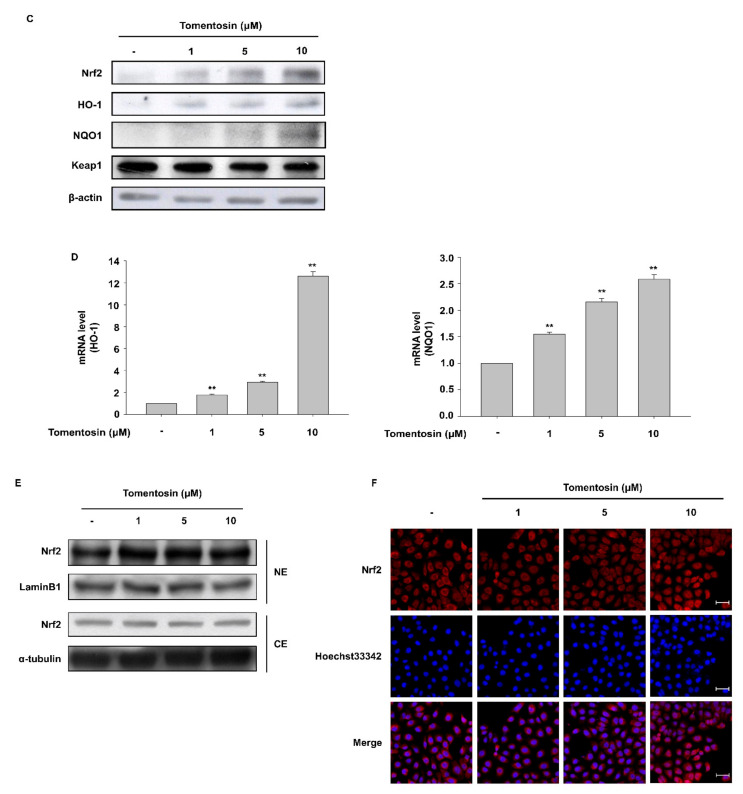
Tomentosin exerts antioxidant activity through activation of the nuclear factor erythroid 2-related factor 2 (Nrf2) signaling pathway. (**A**) 2,2-diphenyl-1-picrylhydrazyl (DPPH) radical scavenging activity of tomentosin. DPPH (0.15 mM) was allowed to react with tomentosin and vitamin C. The data are shown as the mean ± SD of triplicates. (**B**) HaCaT cells were transfected with an antioxidant response element- (ARE-) luciferase reporter and incubated for 4 h. The cells were then incubated with the indicated concentrations of tomentosin for 24 h and subjected to a luciferase reporter assay. The data are presented as the mean ± SD of triplicates. TBHQ was introduced as a positive control. TBHQ, tert-Butyl hydroquinone. (**C**–**F**) HaCaT cells were incubated with 1, 5, or 10 μM tomentosin for 24 h. (**C**) Protein levels of NAD(P)H-quinone oxidoreductase 1 (NQO1), Nrf2, Kelch-like ECH-associated protein 1 (Keap1), and heme oxygenase-1 (HO-1) were analyzed by Western blot. β-actin was introduced as the control for whole-cell lysates. (**D**) mRNA levels of HO-1 and NQO1 were analyzed by qRT-PCR. Glyceraldehyde 3-phosphate dehydrogenase (GAPDH) was used as the control. The results are shown as the mean ± SD of triplicates. ** *p* < 0.01 vs. untreated group. (**E**,**F**) Cells were analyzed by Western blotting (**E**) of nuclear and cytoplasmic fractions and by immunocytochemistry (**F**) to assess the level of Nrf2 nuclear translocation. α-tubulin and lamin B1 were introduced as the controls for cytoplasmic and nuclear protein extracts, respectively. NE, nuclear extracts; CE, cytosolic extracts. Scale bar = 50 μm.

In previous experiments, tomentosin lowered ROS levels in cells, and thus exhibited antioxidant activity. Therefore, we performed a DPPH assay to examine whether tomentosin possesses radical-scavenging activity. However, contrary to our expectation, tomentosin did not reduce DPPH free radicals in vitro (Figure 2A). Next, we examined the effect of tomentosin on Nrf2 signaling, which is one of the main antioxidant signaling pathways in HaCaT cells. In this experiment, tomentosin significantly increased ARE reporter activity in a concentration-dependent manner (Figure 2B). We further examined the expression of Nrf2 and its target genes *HO-1* and *NQO1*. Tomentosin increased the protein levels of Nrf2 and its target genes including *HO-1* and *NQO1* in a concentration-dependent manner (Figure 2C). The mRNA levels of *HO-1* and *NQO1* were also increased by tomentosin treatment (Figure 2D). On the other hand, tomentosin reduced Keap1 protein levels; this protein is involved in mediating the degradation of Nrf2 in the cytoplasm (Figure 2C). In addition, Western blot and immunocytochemistry analyses showed that tomentosin increased the nuclear translocation of the Nrf2 protein (Figure 2E,F). These results indicate that tomentosin exerts antioxidant activity through activation of the Nrf2 signaling pathway.

### 3.3. Tomentosin Induces Nrf2 Activation by Phosphorylating p38 MAPK and JNK in HaCaT Cells

**Figure 3:**
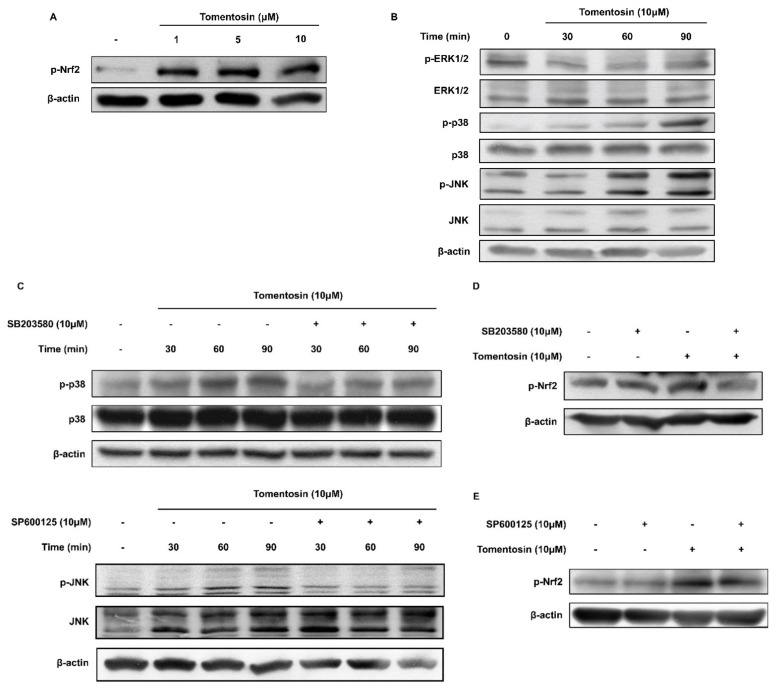
Tomentosin induces the phosphorylation of nuclear factor erythroid 2-related factor 2 (Nrf2) via the p38 and c-Jun *N*-terminal kinase (JNK) mitogen-activated protein kinase (MAPK) pathway. (**A**) HaCaT cells were incubated with 1, 5, or 10 μM tomentosin for 24 h. The treated cells were then harvested and analyzed for Nrf2 phosphorylation by Western blotting. β-actin was introduced as the control for whole cell lysates. (**B**) Cells were treated with 10 μM tomentosin for the indicated time periods (30–90 min). After the treatment period, the cells were collected and analyzed for the phosphorylation of extracellular signal-regulated kinase (ERK), p38 MAPK, and JNK by Western blotting. β-actin was introduced as the control for whole cell lysates. (**C**) Cells were treated with either 10 μM SB203580 or 10 μM SP600125 in addition to treatment with 10 μM tomentosin for the indicated time periods (30–90 min). After the treatment period, the cells were collected and analyzed for the phosphorylation of JNK and p38 MAPK by Western blotting. β-actin was used as the control for whole-cell lysates. (**D**,**E**) Cells were co-treated with 10 μM tomentosin and either (**D**) 10 μM SB203580 or (**E**) 10 μM SP600125 for 24 h. After 24 h, the cells were harvested and analyzed for the phosphorylation of Nrf2 by Western blotting. β-actin was introduced as the control for whole-cell lysates.

To examine the molecular mechanisms of action underlying Nrf2 activation by tomentosin, we investigated the relationship between phosphorylation of MAPKs and Nrf2 using Western blot analysis. Tomentosin treatment increased the phosphorylation levels of Nrf2 (Figure 3A). Additionally, tomentosin induced the phosphorylation of p38 MAPK and JNK in a time-dependent manner but did not show this effect on p42/44 MAPK (Figure 3B). To investigate the involvement of p38 MAPK and JNK in the phosphorylation of Nrf2, we performed Western blot analysis using SB203580 (a p38 MAPK inhibitor) and SP600125 (a JNK inhibitor) and found that treatment with these inhibitors significantly reduced the phosphorylation levels of p38 MAPK and JNK, respectively (Figure 3C). Moreover, as shown in Figure 3D,E, SB203580 and SP600125 attenuated the tomentosin-induced phosphorylation of Nrf2. These data indicate that tomentosin induces Nrf2 phosphorylation through activation of p38 MAPK and JNK.

### 3.4. Tomentosin-Induced ROS Production Mediates Nrf2 Activation

**Figure 4:**
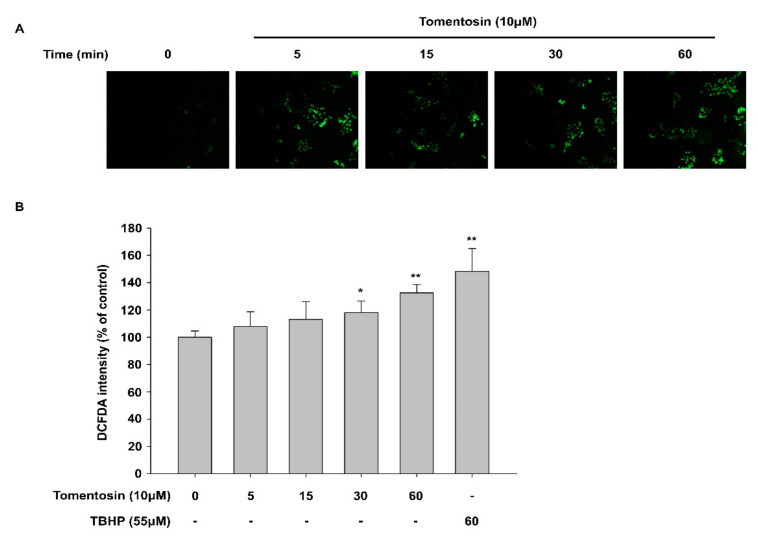

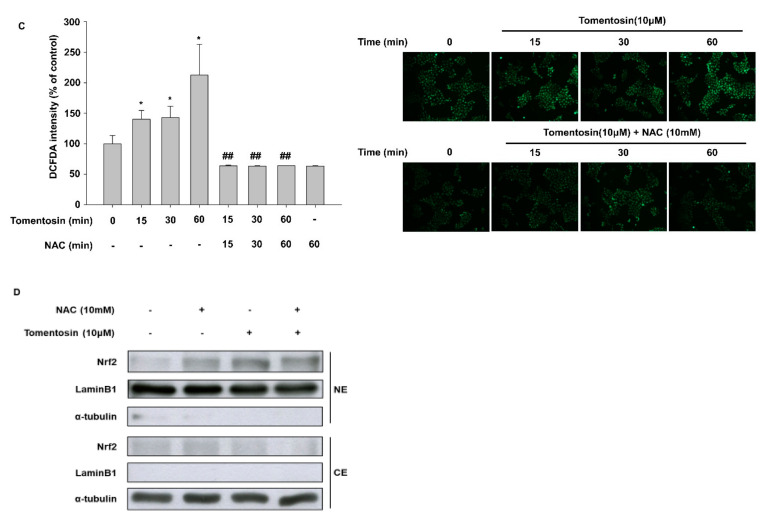
Tomentosin-derived ROS production mediates activation of nuclear factor erythroid 2-related factor 2 (Nrf2). (**A**,**B**) HaCaT cells were incubated with 10 μM tomentosin for the indicated time periods (5–60 min) and treated with 20 μM 2′,7′ dichlorofluorescin diacetate (DCFDA) for 20 min. The cells were then subjected to fluorescence microscopy (Original magnification = 10×) (**A**) or fluorescence intensity analysis (**B**). The results are shown as the mean ± SD of triplicates. * *p* < 0.05 vs. untreated group. ** *p* < 0.01 vs. untreated group. (**C**,**D**) Cells were treated with 10 μM tomentosin in the presence or absence of 10 mM *N*-acetyl-*_L_*-cysteine (NAC) for the indicated time points and then treated with 20 μM DCFDA for 20 min. The cells were subjected to fluorescence intensity analysis (**C, left**) and fluorescence microscopy (**C, right**)(Original magnification = 10×). The results are shown as the mean ± SD of triplicates. * *p* < 0.05 vs. untreated group. ## *p* < 0.01 vs. tomentosin-treated group. (**D**) Cells were treated with 10 μM tomentosin in the presence of 10 mM NAC for 24 h. The treated cells were collected, and the nuclear and cytoplasmic cellular fractions were analyzed by Western blotting. α-tubulin and lamin B1 were introduced as controls for cytoplasmic and nuclear protein extracts, respectively. NE, nuclear extracts; CE, cytosolic extracts.

Previous reports have shown that Nrf2 can be activated by electrophilic stress and mild oxidative stress [19,20]. This suggests that some types of antioxidants can exert their antioxidant activity through the ROS-dependent activation of Nrf2 signaling. Therefore, to elucidate the involvement of ROS in the tomentosin-induced activation of Nrf2 signaling, we investigated the effect of tomentosin treatment on ROS production. Although tomentosin reduced ROS levels when the treatment duration was 6–12 h (Figure 1E,F), it triggered the production of ROS when the treatment duration was shorter than 60 min (Figure 4A,B). This effect of tomentosin on ROS production was also significantly attenuated by *N*-acetyl-*_L_*-cysteine (NAC), a strong antioxidant (Figure 4C). In addition, to examine whether tomentosin-induced ROS activates Nrf2, we analyzed the nuclear translocation of Nrf2 in tomentosin-treated cells in the presence of NAC. While Nrf2 protein was increased in the nuclear fraction of tomentosin-treated cells compared to untreated cells, NAC attenuated the Nrf2 nuclear translocation induced by tomentosin (Figure 4D). These findings indicate that tomentosin activates Nrf2 signaling by inducing low levels of ROS production.

### 3.5. Tomentosin Suppresses B[a]P-Induced AhR Signaling

**Figure 5:**
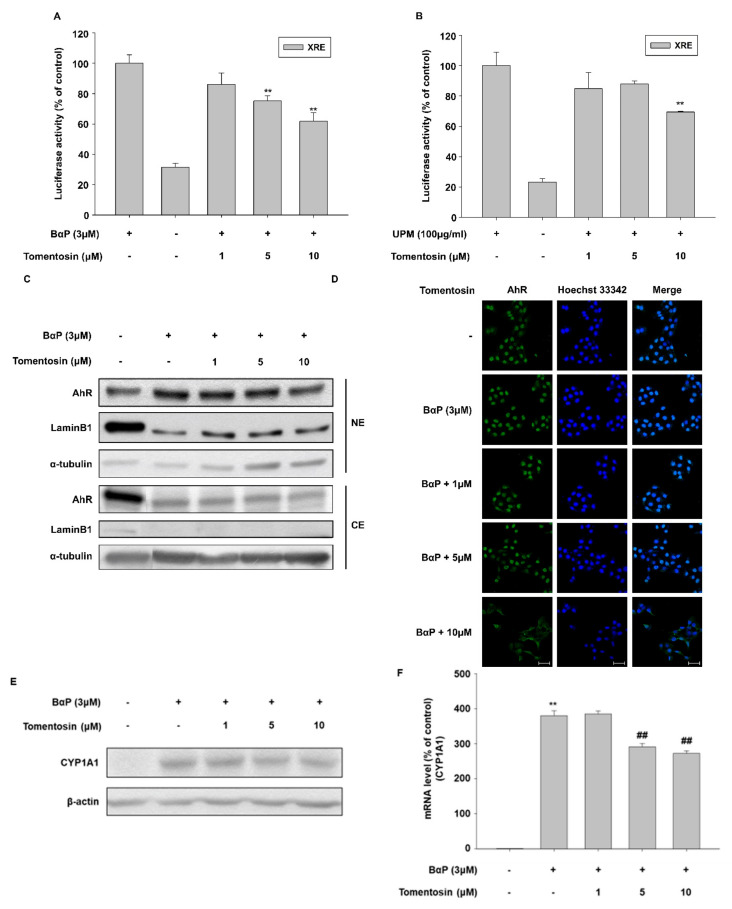
Tomentosin suppresses aryl hydrocarbon receptor (AhR) signaling activated by benzo[a]pyrene (B[a]P). (**A**,**C**–**H**) HaCaT cells were treated with 1, 5, or 10 μM tomentosin in the presence of B[a]P (3 μM) for 24 h. (**B**) HaCaT cells were incubated with 1, 5, or 10 μM tomentosin in the presence of urban particulate matter (UPM) (100 μg/mL). (**A**,**B**) HaCaT cells were transfected with the xenobiotic response element (XRE)-luciferase reporter, and treated with 1, 5, or 10 μM tomentosin in the presence of B[a]P (3 μM) or UPM (100 μg/mL) for 24 h. The cells were then analyzed by a luciferase reporter assay. The results are presented as the mean ± SD of triplicates. (**C**,**D**) HaCaT cells were treated with 1, 5, or 10 μM tomentosin in the presence of B[a]P (3 μM) for 24 h. After treatment, the cells were collected and analyzed by Western blotting (**C**) of the nuclear and cytoplasmic fractions and by immunocytochemical analysis (**D**) to assess AhR nuclear translocation. α tubulin and lamin B1 were introduced as controls for the cytoplasmic and nuclear protein extracts, respectively. NE, nuclear extracts; CE, cytosolic extracts. Scale bar = 50 μm (**E**,**F**) HaCaT cells were treated with 1, 5, or 10 μM tomentosin in the presence of B[a]P (3 μM) for 24 h. (**E**) CYP1A1 protein expression was analyzed by Western blotting. β-actin was introduced as the control for whole-cell lysates. (**F**) *CYP1A1* mRNA levels were analyzed by qRT-PCR; glyceraldeh yde 3-phosphate dehydrogenase (GAPDH) was introduced as the control. The results are presented as the mean ± SD of triplicates. ** *p* < 0.01 vs. untreated group. ## *p* < 0.01 vs. B[a]P-treated group.

B[a]P is a well-investigated AhR ligand [21,22]. We investigated the effects of tomentosin on B[a]P-induced AhR signaling by analyzing the following: the activity of a xenobiotic-response element (XRE)-luciferase reporter, AhR nuclear translocation, and cytochrome P450 1A1 (CYP1A1) expression. Tomentosin treatment reduced B[a]P-induced XRE-luciferase activity in a concentration-dependent manner (Figure 5A). In this experiment, we also used UPM, which is known as a ligand of AhR. Similar to B[a]P, tomentosin reduced UPM-induced XRE-luciferase activity (Figure 5B). In addition, tomentosin inhibited B[a]P-activated AhR nuclear translocation, as evidenced by Western blotting data (Figure 5C) and immunocytochemistry (Figure 5D). Furthermore, while protein and mRNA levels of CYP1A1 were increased by B[a]P treatment, tomentosin reduced the B[a]P-induced upregulation of CYP1A1 expression (Figure 5E,F). These results indicate that tomentosin also regulates B[a]P-induced AhR signaling.

**Figure 6:**
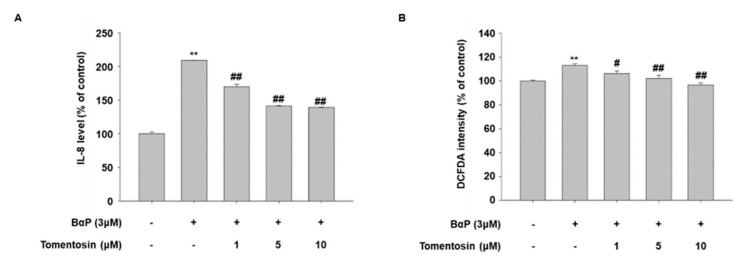
Tomentosin reduces the production of intracellular interleukin (IL)-8 and reactive oxygen species (ROS) induced by benzo[a]pyrene (B[a]P). (**A**,**B**) HaCaT cells were treated with 1, 5, or 10 μM tomentosin in the presence of B[a]P (3 μM) for 24 h. (**A**) After treatment, the cells were subjected to enzyme-linked immunosorbent assay (ELISA) for the quantitation of IL-8. The data were confirmed in at least three independent experiments; the results are shown as the mean ± SD of duplicates. (**B**) After treatment, the cells were incubated with 20 μM dichlorofluorescin diacetate (DCFDA) for 20 min and subjected to fluorescence intensity analysis. The data show the mean ± SD of triplicates. ** *p* < 0.01 vs. untreated group. # *p* < 0.05 vs. B[a]P treated group. ## *p* < 0.01 vs. B[a]P treated group.

It is well known that B[a]P induces the production of ROS and IL-8 (a proinflammatory cytokine) through AhR activity in human keratinocytes [12,23]. In addition, we found that tomentosin suppresses B[a]P-induced AhR signaling. Therefore, we investigated the effect of tomentosin on the production of IL-8 and ROS in HaCaT cells. As shown in Figure 6A,B, tomentosin reduced the B[a]P-induced production of IL-8 and ROS. These results indicate that tomentosin can exert an antagonizing effect against B[a]P-induced cellular changes.

## 4. Discussion

The skin is the outermost organ of our body and plays as a barrier protecting the body from external stress. The skin is composed of three layers, namely the epidermis, dermis, and subcutis. The epidermis is the upper layer of the skin [24] and is in direct contact with environmental stressors such as air pollutants [25]. As keratinocytes constitute 95% of the epidermis in human skin, the condition of keratinocytes is crucial for maintaining the barrier function of the skin [26]. In this study, we demonstrated a novel protective effect of tomentosin on HaCaT, normal human keratinocyte cells.

In this present study, we found that tomentosin shows its antioxidant activity by activating Nrf2-mediated signaling in human epidermal keratinocytes. Tomentosin upregulated the expression of Nrf2 and its target genes such as *HO-1* and *NQO1*. In addition, B[a]P-induced AhR signaling was suppressed by tomentosin treatment. Tomentosin inhibited B[a]P-activated nuclear translocation of AhR and decreased B[a]P-induced upregulation of *CYP1A1* expression. These data indicate that tomentosin exerts its protective activity towards cells by activating Nrf2 signaling and inhibiting AhR signaling.

Under basal conditions, Nrf2 forms a complex with Keap1 and Cul3 in the cytoplasm. Keap1 mediates the ubiquitination and proteasomal degradation of Nrf2 [27]. However, when cells are exposed to mild oxidative stress, the cysteine residues of Keap1 are oxidized, leading to a conformational change in the complex [28]. At this stage, Nrf2 dissociates from the complex and is activated and phosphorylated by intracellular molecules such as Akt and MAPKs [9]. Finally, the free Nrf2 moves to the nucleus and initiates the transcription of its target genes [29]. In this study, the expression of the Nrf2 repressor protein Keap1 was downregulated by treatment with tomentosin. In addition, tomentosin-induced Nrf2 phosphorylation was suppressed by SP600125 (a JNK MAPK inhibitor) and SB203580 (a p38 MAPK inhibitor). These data indicate that tomentosin induces Nrf2 signaling by phosphorylating Nrf2 through the activation of JNK and p38 MAPK. Further, our results also indicate that tomentosin-induced downregulation of Keap-1 contributes to Nrf2 activation.

Interestingly, in this study, we found that treatment with tomentosin for up to 60 min increased ROS production. However, treatment for ≥6 h (6–12 h) resulted in a reduction in ROS levels. To examine the role of tomentosin-induced ROS production in the activation of Nrf2 signaling, we examined the nuclear translocation of Nrf2 using *N*-acetyl-*_L_*-cysteine (NAC), which is a strong antioxidant. Co-treatment with NAC attenuated the tomentosin-induced nuclear translocation of Nrf2. This result suggests that ROS produced due to short-term tomentosin treatment contributes to Nrf2 activation by inducing the nuclear translocation of Nrf2.

AhR is a ligand-dependent transcription factor that controls the expression of xenobiotic-metabolizing enzymes [30]. It is activated in response to external environmental stressors such as polycyclic aromatic hydrocarbons (PAHs), UPM, B[a]P, and dioxins [31,32,33]. When a ligand interacts with AhR, the AhR–ligand complex moves to the nucleus and binds to the XRE region. The activated AhR then increases the expression of xenobiotic-metabolizing enzymes such as cytochrome P450 1A1 (CYP1A1) [30]. AhR signaling has also been known to cause cell and tissue damage [34,35]. In this study, we demonstrated that tomentosin treatment inhibited the B[a]P-induced AhR signaling pathway. Tomentosin suppressed the following B[a]P-induced effects: AhR nuclear translocation, *CYP1A1* expression, and the production of ROS and IL-8. These results suggest that tomentosin ameliorates cell damage induced by B[a]P through the inhibition of the AhR signaling pathway.

Oxidative stress has been linked to various types of diseases [5,36,37]. Previous studies have reported on several natural compounds with antioxidant and cell-protective activities [38,39]. For example, cynaropicrin induces the translocation of AhR into the nucleus (by binding to it) and activates the Nrf2 signaling pathway; this protects cells from ROS-mediated oxidative damage [40]. In addition, while resorcinol inhibits AhR, it activates the Nrf2 pathway and exerts antioxidant activity in an AhR-independent manner [41]. In this study, we found that tomentosin showed a mechanism of action similar to that of resorcinol. However, in contrast to resorcinol, tomentosin induced Nrf2 signaling by producing ROS and activating p38 MAPK and JNK signaling.

As discussed above, we demonstrated that tomentosin exerted antioxidant and cell-protective effects in human epidermal keratinocytes. These effects were exerted by activating Nrf2 signaling (by inducing the production of low levels of ROS), and p38 MAPK and JNK pathways, and by suppressing AhR signaling (by blocking the nuclear translocation of AhR). Our findings suggest that tomentosin and tomentosin-containing aromatic medicinal plant species could be used to ameliorate skin disorders caused by oxidative stress or air pollutants such as B[a]P.

## 5. Conclusions

**Figure 7:**
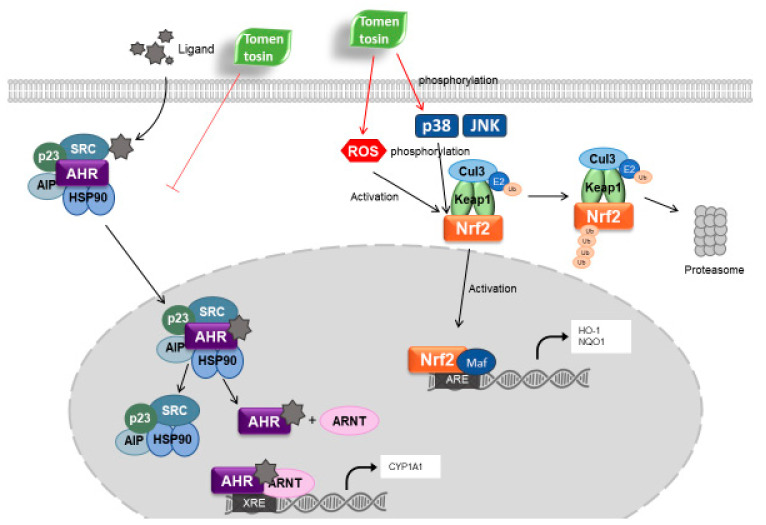
Action mechanism of tomentosin in the AHR/Nrf2-mediated signaling. Tomentosin activates Nrf2 signaling through activation of p38 MAPK and JNK as well as ROS production. Tomentosin also inhibits AHR signaling by suppressing nuclear translocation of AHR. Red line: Action step of tomentosin.

In this study, we demonstrated that tomentosin upregulates the Nrf2 antioxidant pathway through ROS production as well as by the activation of JNK and p38 MAPK. In addition, tomentosin suppresses B[a]P-activated AhR signaling and inhibits the production of IL-8 and ROS induced by B[a]P. The mechanism of tomentosin in the AHR/Nrf2-mediated signaling is presented in Figure 7. These data suggest that tomentosin could be applied therapeutically for oxidative stress-related skin disorders.

## Data Availability

The data presented in this study are available in the article.
